# Anther development in tribe Epidendreae: orchids with contrasting pollination syndromes

**DOI:** 10.7717/peerj.4383

**Published:** 2018-02-27

**Authors:** Benjamín Valencia-Nieto, Victoria Sosa, Judith Márquez-Guzmán

**Affiliations:** 1 Laboratorio de Desarrollo en Plantas, Departamento de Biología Comparada, Facultad de Ciencias, Universidad Nacional Autónoma de México, CDMX, México; 2 Biología Evolutiva, Instituto de Ecología AC, Xalapa, Veracruz, México

**Keywords:** *Chysis*, Incumbency, Epidendreae, Anther, Development, Orchidaceae, Pollination

## Abstract

**Background:**

Epidendreae is one of the most diverse tribes among the orchids with remarkable variation in life form, floral morphology and pollination syndromes. Its circumscription was recently revised and subtribes Agrostophyllinae and Calypsoinae were transferred into this tribe. One of the principal floral characters utilized in classification of orchids is the incumbency or bending of the column. This study records and compares late stages of anther, column and lip development, and discusses anther characters in fifteen representative taxa of five of the six subtribes in Epidendreae with respect to classification and pollination biology.

**Methods:**

A series of late floral stages were sampled and fixed for examination under scanning electron microscope.

**Results:**

Anther incumbency or bending in this group varies from 90° to almost 180°. Incumbency in the late stages of development is reached in Bletiinae, Ponerinae, Pleurothallidinae and Laeliinae whereas incumbency is reached early in its development in *Corallorhiza* and *Govenia* of Calypsoinae.

**Discussion:**

Our observations indicate that the position of *Chysis* in subtribe Bletiinae needs revision based on differences in a number floral, and in particular of anther characters; and that *Coelia* only shares the early anther incumbency with Calypsoinae members, but not the rest of floral and anther characters. Anatomical characters such as crystals around the actinocytic stomata on the anther cap and sugar crystals in Laeliinae; lack of rostellum in Bletiinae; coalescent anther with the column, lack of trichomes and papillae on lip keels, and underdeveloped rostellum in *Chysis*; a mechanism by which the anther cap comes off (it is joined with the grooved lip by a claw) in *Isochilus* are all related to pollination syndromes and reproductive biology.

## Introduction

Epidendroideae is an orchid subfamily with more than 21,000 taxa, the largest in this group with approximately 76%–80% of the total species in the family (Freudenstein & Chase, 2015; Givnish et al., 2015). Within this subfamily, the tribe Epidendreae has been recently identified as an example of flowering plant radiations associated with epiphytism and with anther characters related to pollinator specificity (Freudenstein & Chase, 2015). Epidendreae includes one of the largest genera in the Neotropics, the genus *Epidendrum* L (Hágsater & Soto-Arenas, 2005; Pinheiro et al., 2009; Pinheiro & Cozzolino, 2013) and also a diverse group composed of minute plants, the Pleurothallidinae (Pridgeon, Solano & Chase, 2001; Blanco & Barboza, 2005; Damon & Roblero, 2007; Karremans et al., 2015; Karremans, 2016). Plants in this tribe display remarkable variation in life form, floral morphology and pollination syndromes (Van Der Pijl, Dodson & Flowers, 1969; Van den Berg, 2005; Jersáková, Johnson & Kindlmann, 2006; Freudenstein & Chase, 2015): for instance the showy insect-pollinated laelias and cattleyas (Halbinger & Soto-Arenas, 1997; Borba & Braga, 2003; Van den Berg et al., 2009); the fragrant encyclias and prosthecheas, usually bee pollinated and exclusively pollinated by wasps respectively (Higgins, 2003); *Chysis* Lindl. with large fusiform bulbs and fleshy-waxy flowers probably pollinated by different bees (Dressler, 1993; Soto-Arenas, 2005a; Soto-Arenas & Solano-Gómez, 2007; Soto-Arenas, 2008a); *Isochilus* R.Br. with reed-like stems, inconspicuous bulb base and tiny, racemose tubular hummingbird-pollinated flowers (Salazar, 2005; Soto-Arenas, 2008b; Siegel, 2011); the terrestrial *Bletia* Ruiz & Pav. with deceit pollination (Sosa, 2007; Salazar, Chavez-Rendon & Jiménez-Machorro, 2016), the mostly autopollinated and mycoheterotrophic *Hexalectris* Raf. (Fowler, 2005; Sosa et al., 2016), as well as the insect pollinated genera *Calypso* Salisb. and *Govenia* Lindl. (Whigham & McWethy, 1980; Van Der Cingel, 2001; García-Cruz & Sosa, 2005; Freudenstein, 2005a; Freudenstein, 2005b; Freudenstein, 2005c; Freudenstein, 2005d; Pansarin, 2008; García-Cruz et al., 2009; Tuomi et al., 2015). According to current classification of Orchidaceae derived from recent phylogenies, tribe Epidendreae is composed of six subtribes (Agrostophyllinae, Calypsoinae, Bletiinae, Ponerinae, Pleurothallidinae, Laeliinae) (Van den Berg et al., 2005; Freudenstein & Chase, 2015; Chase et al., 2015; Givnish et al., 2015) (Table 1). With the recent addition of the Asian *Earina* and the North American *Calypso* the tribe is no longer exclusive of the Neotropics as was previously considered by Van den Berg et al. (2005) and Van den Berg et al. (2000).

**Table 1:** Subtribes in Tribe Epidendreae, their distribution, diversity and pollination syndromes. The representative taxa studied here are indicated.

		Calypsoinae		Bletiinae			
Subtribe	Agrostophyllinae		Coeliinae	Chysinae	Ponerinae	Pleurothallidinae	Laeliinae
Distribution	Old World	Old and New World	New World	New World	New World	New World	New World
Included and observed in this study.	None	Co*rallorhiza maculata, Govenia mutica*	*Coelia triptera*	*Bletia purpurea, Chysis bractescens, Chysis limminghei, Chysis laevis*	*Isochilus major, Ponera juncifolia*	*Stelis ciliaris, Specklinia digitale*	*Laelia speciosa, Oestlundia ligulata, Prosthechea squalida*, *Encyclia microbulbon*
Pollinia number	8 and 4	4	8	8	4 and 6	2, 4, 6 and 8	2, 4, 6, 8 and 12
Pollinators or pollination syndromes.	Halictid bees and autogamy (Pridgeon et al., 2005)	Bumblebees, hover flies, empididae flies, mosquitoes, bees, facultative autogamy (Whigham & McWethy, 1980; Van Der Cingel, 2001; Freudenstein, 2005a; Freudenstein, 2005b; Freudenstein, 2005c; Freudenstein, 2005d; García-Cruz & Sosa, 2005; Pansarin, 2008; García-Cruz et al., 2009; Tuomi et al., 2015)	Unknown	Various kinds of bees, bumblebees, autogamy (Van Der Cingel, 2001; Fowler, 2005; Soto-Arenas, 2005a; Soto-Arenas & Solano-Gómez, 2007; Sosa, 2007; Sosa et al., 2016; Salazar, Chavez-Rendon & Jiménez-Machorro, 2016)	Hummingbirds, small bees and wasps (Salazar, 2005; Soto-Arenas, 2005a; Siegel, 2011)	Diptera (Pridgeon, Solano & Chase, 2001; Blanco & Barboza, 2005; Jersáková, Johnson & Kindlmann, 2006; Damon & Roblero, 2007; Karremans et al., 2015)	Bees, wasps, birds, butterflies, moths, diptera (Van Der Pijl, Dodson & Flowers, 1969; Dressler, 1993; Halbinger & Soto-Arenas, 1997; Borba & Braga, 2003; Higgins, 2003; Soto-Arenas & Solano-Gómez, 2007; Soto-Arenas, 2008a; Soto-Arenas, 2008b; Van den Berg et al., 2009)

In orchids in general, including Epidendreae, the most noticeable element of the flower is the labellum, an element of the inner whorl of tepals, showing evident diversity of form and color (Rudall & Bateman, 2004; Rudall, 2007). Another striking element is the column, formed by the androecium and stigma/style with a single stamen with pollen grains variously aggregated into pollinia (Dressler, 1981). Commonly, the position of the labellum and column are determinant for forcing the pollinator to approach the flower in a certain way so that pollinaria are placed on the pollinator; pollinia may move or change orientation after removal by the pollinator in a way to successfully pollinate the next flower visited (Van Der Pijl, Dodson & Flowers, 1969; Peter & Johnson, 2005). The pollinia have reached remarkable complexity in some orchid groups, including Epidendreae (Dressler, 1993): they usually have several appendages such as the viscidium (part of the rostellum, the section of the median stigma or tissue separating the anther from the fertile stigma), the sticky pad, a structure that adheres to pollinators (Dressler, 1993) and in some species only a liquid on the rostellum, the viscarium allows pollinators to touch pollinaria (Dressler & Salazar, 1991; Hágsater et al., 2005). The caudicles are a slender, mealy or elastic extension of the pollinaria, or a mealy portion at one end of the pollinarium, produced within the anther and by which the pollinia attach to the viscidium or to pollinators (Dressler, 1981; Dressler, 1993; Chase et al., 2003). Another important feature of the anther is the anther cap that covers the pollinarium, in most species, this cap drops off the pollinarium following its removal by a pollinator, but in a few species the anther cap embraces the pollinia, for some time before the anther cap drops off (Dressler, 1981; Peter & Johnson, 2005).

Based on phylogenetic research it is considered that the primitive condition of the anther is erect and parallel to the axis of the column as in most Orchidoideae; whereas most Epidendroideae have the derived condition, an erect anther in the early bud development, bending 90° to 120° by column elongation, so that it comes to rest, like a cap, on the apex of the column. The term incumbent is used for this condition (Dressler, 1981; Dressler, 1993; Chase et al., 2003; Freudenstein & Chase, 2015) and is considered the most important defining character of the subfamily Epidendroideae (Kurzweil, 1987; Dressler, 1981; Dressler, 1993; Arditti, 1992; Freudenstein, Harris & Rasmussen, 2002; Freudenstein & Chase, 2015).

Here we focus on recording and comparing the late stages in the ontogeny of the anther in representative species of tribe Epidendreae, considering five out of the six subtribes. The incumbent anther has been identified as the key synapomorphy for the subfamily Epidendroideae; however, there are two general ways of reaching the inflexion of the anther in this group, one way by the reorientation of growth in the early ontogenetic stages of the anther (called vandoid morphology), and the other, as the result of elongation of the column and the inflexion of the mature anther during the late stages of development (Freudenstein, Harris & Rasmussen, 2002; Freudenstein & Chase, 2015). The degree of inflexion varies among species, and reversal has been identified in several bird-pollinated species (Rasmussen, 1982; Freudenstein, Harris & Rasmussen, 2002). For instance, in *Coelia* it has been determined that it exhibits early anther inflexion, which is characteristic of the vandoid morphology; but it lacks superposed pollinia and pollinium stalks, both are also key characteristics of this syndrome (Freudenstein & Chase, 2015; Freudenstein, Yukawa & Luo, 2017).

Kurzweil (1987) set the bases of the knowledge on column and anther development in this group: his work consisted basically of morphological descriptions, using scanning electron microscopy, of the early stages to anthesis of few species of different genera belonging to subfamily Epidendroideae; and based on differences on column and anther characters he reached taxonomic conclusions. Later Freudenstein (1994a), Freudenstein (1994b) and Freudenstein, Harris & Rasmussen (2002) focused on the incumbency of the anther in the Vandoid orchids, detailing the process by which this group of Epidendroideae orchids develops inflexion, characterizing the vandoid morphology. Recently, Valencia-Nieto, Espinosa-Matias & Márquez-Guzmán (2011) and Valencia-Nieto, Sosa & Márquez-Guzmán (2016) provided detailed descriptions of the late ontogeny of column and anther in representative taxa of subtribe Laeliinae, using this character to hypothesize the phylogenetic position of the controversial genus *Microepidendrum*. Moreover, Freudenstein & Chase (2015) analyzed a few anther characters using the most recent phylogenetic hypothesis to understand patterns of diversification, looking for correlations between changes in specific characters and species diversity focusing in the Vandoid morphology (superposed pollinia, early anther inflexion and cellular pollinium stalk). However, these anther characters are not present among the most diverse Epidendreae subtribes, and certain flower characteristics have been identified as being related with pollinator specificity, thus creating an opportunity to conduct detailed studies of late anther morphology and analyze these characters.

This study records and compares late stages of anther development in fifteen representative taxa, belonging to five of the six subtribes in Epidendreae. The late ontogeny of the column is observed with emphasis on the lip, column and anther. These characters are discussed in light of classification and pollination syndrome.

## Materials and Methods

*Field study permissions–* Biological samples of flowers and buds were obtained from the living collection maintained by the Botanical Garden of the Instituto de Biología, UNAM and the botanical garden “Francisco Javier Clavijero” of the Instituto de Ecología, A.C. (INECOL). Access to the material was obtained under the terms of scientific permits MX-JB-008-DF and VER-FLO-228-09-09. The Mexican species of Orchidaceae are under special protection (Norma Oficial Mexicana, NOM-059-ECOL-2010, Secretaría de Medio Ambiente y Recursos Naturales, Diario Oficial de la Federación 30 December 2010, Mexico, DF) and the botanical gardens hold individuals of every collected taxon. Data on vouchers is indicated in Table S1.

**Figure 1:**
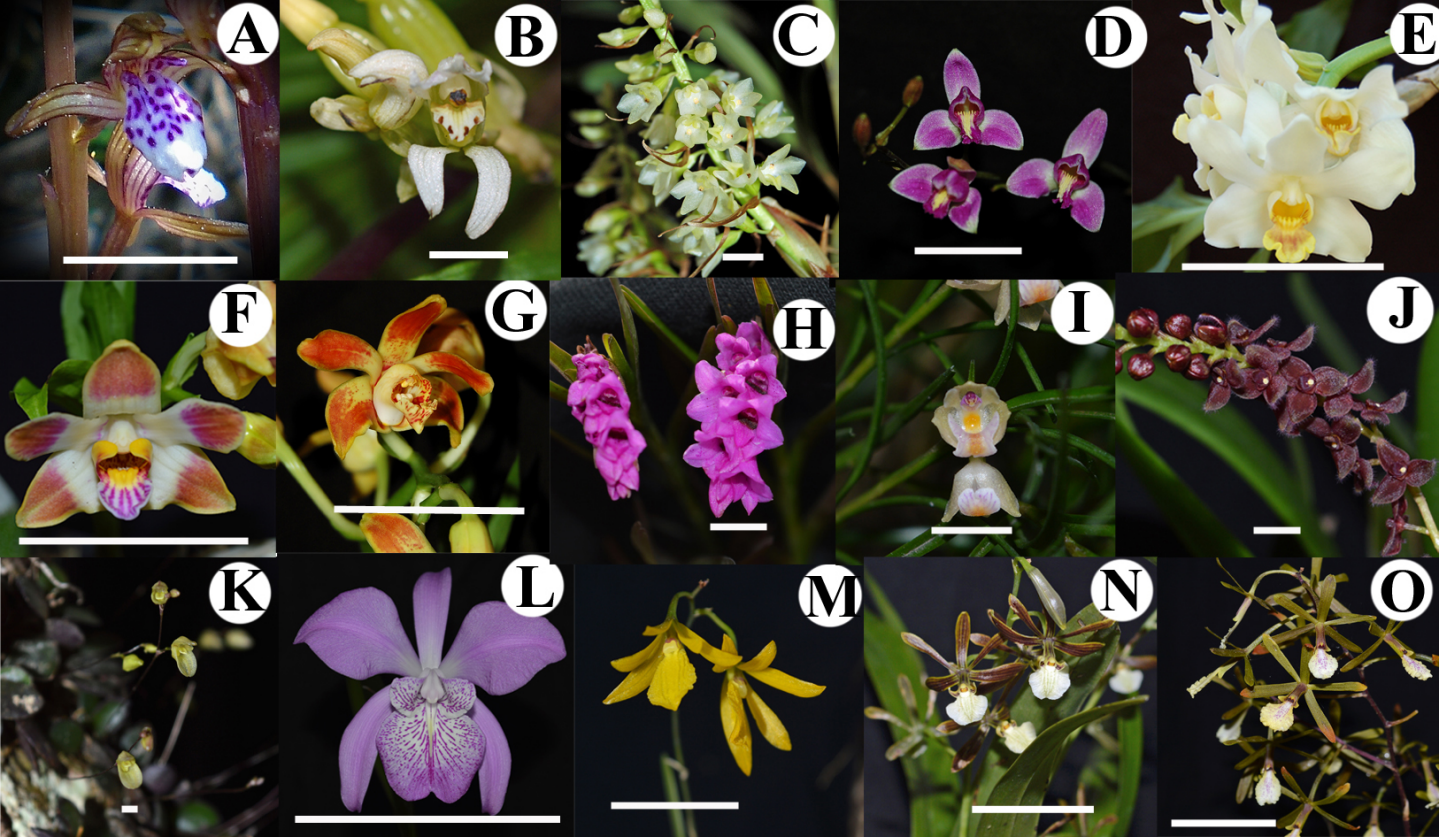
Studied species of Tribe Epidendreae. (A) *Corallorhiza maculata* (Calypsoinae). (B) *Govenia alba* (Calypsoinae). (C) *Coelia triptera* (Coeliinae)*.* (D) *Bletia purpurea* (Bletiinae)*.* (E) *Chysis bractescens* (Bletiinae)*.* (F) *Chysis limminghei* (Bletiinae). (G) *Chysis laevis* (Bletiinae)*.* (H) *Isochilus major* (Ponerinae)*.* (I) *Ponera juncifolia* (Ponerinae)*.* (J) *Stelis ciliaris* (Pleurothallidinae)*.* (K) *Specklinia digita* l*e* (Pleurothallidinae). (L) *Laelia speciosa* (Laeliinae). (M) *Oestlundia ligulata* (Laeliinae). (N) *Prosthechea squalida* (Laeliinae). (O) *Encyclia microbulbon* (Laeliinae). Scale Bars: A = 1.5 cm. B = 1 cm. C = 1.2 cm. D = 5 cm. E = 9 cm. F = 5.5 cm. G = 6.5 cm. H = 1 cm. I = 5 mm. J = 4.5 mm. K = 3 mm. L = 14 cm. M = 1.4 cm. N = 2 cm. O = 3.5 cm.

***Sampling*–**Representative taxa in the subtribes Calypsoinae, Bletiinae, Ponerinae, Pleurothallidinae and Laeliinae of the Epidendreae tribe were selected (Table 1, Fig. 1). Flowers and buds from the following species were studied: *Corallorhiza maculata* (Raf.) Greene (Calypsoinae, Fig. 1A), *Govenia alba* A. Rich. & Galeotti (Calypsoinae, Fig. 1B), *Coelia triptera* (Sm.) Mutel (Calypsoinae, Fig. 1C), *Bletia purpurea* (Lam.) DC. (Bletiinae, Fig. 1D), *Chysis bractescens* Lindl. (Bletiinae, Fig. 1E), *Chysis limminghei* Linden & Rchb.f. (Bletiinae, Fig. 1F), *Chysis laevis* Lindl. (Bletiinae, Fig. 1G), *Isochilus major* Cham. & Schltdl. (Ponerinae, Fig. 1H), *Ponera juncifolia* Lindl. (Ponerinae, Fig. 1I), *Stelis ciliaris* Lindl. (Pleurothallidinae, Fig. 1J*), Specklinia digitale* (Luer) Pridgeon & M.W. Chase (Pleurothallidinae, Fig. 1K), *Laelia speciosa* (Kunth) Schltr. (Laeliinae, Fig. 1L), *Oestlundia ligulata* (La Llave & Lex.) Soto-Arenas (Laeliinae, Fig. 1M)*, Prosthechea squalida* (Lex.) Soto Arenas & Salazar (Laeliinae, Fig. 1N) and *Encyclia microbulbon* Schltr. (Laeliinae, Fig. 1).

***Fixation and processing of tissues*–**Three late floral developmental stages were selected: flowers in anthesis and/or large buds prior to anthesis located at the base of inflorescences; intermediate stages of buds not completely developed located in the middle of inflorescences; and early stages of small buds at the apex of inflorescences. When sufficient individuals were available, three plants were randomly chosen, but in some cases only one plant was available. All buds/flowers were fixed in FAA (formaldehyde, 95% ethanol, glacial acetic acid, distilled water 1 : 5 : 0.5 : 3.5, v/v).

***Scanning electron microscope*** **(SEM)–**Characters were observed under the scanning electron microscope (SEM). Dissected samples were dehydrated in a graded ethanol series (30, 75, 80, 96, 100 and 100% v/v), before critical-point drying with a CPD-030 Bal-tec, and sputter-coating with gold. Observations were performed with a SEM model JSM-5310 LV; JEOL, Tokyo.

## Results

Table 2 summarizes the main anther, column and lip features for the studied taxa of five of the six subtribes in tribe Epidendreae.

**Table 2:** Main features (anther, column and lip development characters) observed.

Late develop-ment charac-ters	Subtribes and the key taxa observed on Epidendreae														
	Calypsoinae			Bletiinae				Ponerinae		Pleurothallidinae		Laeliinae			
	*Corallo-rhiza maculata*	*Govenia mutica*	*Coelia triptera*	*Bletia purpu-rea*	*Chysis brac-tescens*	*Chysis limminghei*	*Chysis laevis*	*Isochilus major*	*Ponera juncifo-lia*	*Stelis ciliaris*	*Speckli-nia digitale*	*Laelia speciosa*	*Oestlundia ligulata*	*Prosthechea squalida*	*Encyclia microbulbon*
Column bending (Fig. 2)	Begins straight ends very arched (apical part pronounced bent over itself)	Begins straight ends arched (apical part pronounced bent over itself)	Always straight	Begins straight ends arched	Begins straight ends arched	Begins straight ends arched	Begins straight ends arched	Begins straight ends slightly arched	Begins straight ends slightly arched	Always straight	Begins straight ends slightly arched	Begins straight ends slightly arched	Always straight	Always straight	Always straight
Column wings (Fig. 2)	Absent	Present	Absent	Present	Absent	Absent	Absent	Absent	Absent	Absent	Present	Absent	Absent^a^	Absent	Absent
Incumbent anther (degree from the original position. Fig. 2)	170°	170°	90°	170°	170°	170°	170°	180°	90°	170°	170°	140°	40°	90°	180°
Early inflexing anther (Fig. 2)	Present	Present	Present	Absent	Absent	Absent	Absent	Absent	Absent	Absent	Absent	Absent	Absent	Absent	Absent
Rostellum (Fig. 3)	Present	Present	Present	Present	Present	Present	Only vestigial and in some cases absent	Present	Present	Present	Present	Present	Present	Present	Present
Anther epider-mis ornamentation (Fig. 4)	Smooth	Smooth	Smooth	Slightly striate	Conspicuously striate	Striate	Striate	Smooth	Smooth to slightly striate	Striate slightly corrugate	Densely striate	Striate	Slightly striate	Slightly striate	Conspicuously striate
Type of stomata in anther (Fig. 4)	Absent	Absent	Actino-cytic type	Actino-cytic type	Actino-cytic type	Actino-cytic type	Actinocy-tic type	Actinocy-tic type	Actino-cytic type	Absent	Absent	Actinocy-tic type	Absent	Actinocytic typr	Semi-actinocy- tic type
Stipe (Fig. 5)	Hamulus	Tegula	Absent	Absent	Absent	Absent	Absent	Absent	Absent	Absent	Absent	Absent	Absent	Absent	Absent
Pollinia numbers (Fig. 5)	4	4	8	8	8	8	8	4	4	2	2	8	4	4	4
Pollinia form (Fig. 5)	Equal	Equal	Equal	Equal	Une-qual	Une-qual	Unequal	Equal	Equal	Equal	Equal	Unequal	Equal	Equal	Equal
Number of caudicles in pairs (Fig. 5)	1	1 (irregular mass of cells)	2	2	1	1	Absent	1	2	Absent	1	2	Absent	2	2
Number of lip keels (Fig. 6)	2	5	0	3 cen-tral 4 accesso-ries	5 central	5 central	3 central 6–8 accesso-ries	0	0	0	2	3	1 central 6 accessories	3 central	2 central
Lip tricho-mes or papillae (Fig. 6)	Absent	Papillae	Absent	Absent	Tricho-mes	Tricho-mes	Absent	Absent	Absent	Tricho-mes	Absent	–	Absent	Trichomes	Absent
Develop-ment of the column and the lip	Free lip	Free lip but, articulated by a column foot	Free lip but, attached to the base by a column foot	Free lip	Free lip but, articulated by a column foot	Free lip but, articulated by a column foot	Free lip but, articulated by a column foot	Free lip but, articulated by a column foot	The lip base attached by a column foot, rest free	Free lip	Free lip but, articulated by a column foot	Free lip	Lip partially fused with the column (1/3 from the base)	Lip basally fused to the column	Lip basally connate to the column
Flower diameter (cm)	1.5	1	1.2	5	9	5.5	6.5	1	.5	.45	.3	14	1.4	2	3.5
lip length × wide (mm)	–	–	5–5.5 ×3.8–4.1	–	25–35 × 28–40	17 × 13	25 × 14	–	–	.9 × .7	1.5–2 ×0.6–1	40–70 × 35–50	3–4 × 2	10–14	–
Anther length × wide (mm)	–	–	1 × 1.3	–	3.3 × 3.3	3.3 × 3	2.5 × 2	–	–	.32 × .3	0.35–0.5 ×0.3–0.4	5–6	1.8 × 1.5	0.75 × 1.1	2 × 1.6
Column length × wide (mm)	–	–	1.9–2.1 ×2.3–2.8	–	15–18 × 8–9	9 × 4.2	15 × 7	–	–	.7 × .25	1.3–2 × 0.8	33–36 × 7–9	4–6 × 5.5	6–6.5 ×2.3–2.5	6–9.5 × 2.3
Ovary length × wide (mm)	–	–	6–9 ×4.5–5	–	15–25 × 5-	12–20 ×3–6	15 × 3.2	–	–	–	0.7–2 × 0.4	–	11–15	11–15 × .4-.6	21–30 × 1.5

**Notes.**

a Soto-Arenas (2008c) mentions the presence of porrect wings in this species, however in our observations what he describes is interpreted as the apical part of the clinandrium.

### Subtribe Calypsoinae: *Corallorhiza maculata*

**Figure 2:**
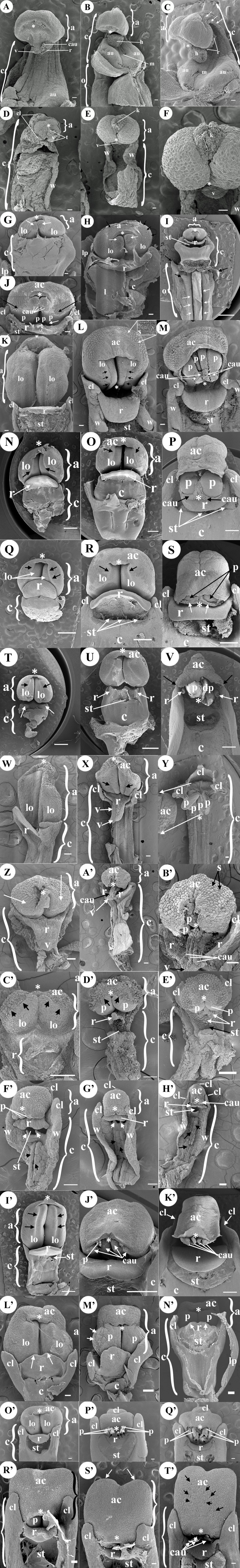
Development micrographs of anther. Late stages of development (column and anther) of *Corallorhiza maculata* (A, B, C). (A) Early incumbent anther. (B) Grown incumbent anther. (C) Lateral view bent over itself column and the incumbent anther. *Govenia alba* (D, E, F) (D) Early** incumbent anther, column erect (same width throughout its length) and small developing wings. (E) Grown-up noticeably inflected anther. (F) Prominent keel in the middle of anther, just below the apical part of tegula (viscidium shield). *Coelia triptera* (G, H, I, J) (G) Early incumbent anther (H) Intermediate late stage incumbent anther. (I) Anther totally inflected, column hexagon-shaped, base joins the ovary which has pointed projections (black arrows) and with large grooves that cross longitudinally (white arrows). (J) Close-up of anther at anthesis, black arrows point the limit between anther cap and each pollinium respectively (two pairs of granulose caudicles exposed at apex). *Bletia purpurea* (K, L, M); (K) Early erect anther, slightly sub quadrate, bilobate with a trapezoid apex. (L) Intermediate late stage, anther inflected, the cucullate anther cap with actinocytic stomata embedded in its upper part (white arrows, close-up in the inset and Fig. 4B). (M) Anthesis, anther inflected, anther cap fully developed, pollinia (four upper visible), caudicles in the apex, at sides column wings. *Chysis bractescens* (N, O, P) (N) Early anther, erect, round shaped, bilobate with longitudinal lines marked on its epidermal tissue (black arrows). (O) Intermediate late stage anther, more quadrate and slightly less erect (apex with some degree of inflection), anther cap visible. (P) Galeate anther totally inflected, cucullate anther cap. A pair of pollinia exposed, with two laminar caudicles (one pair, black arrows) which surround the pollinia. *Chysis limminghei* (Q, R, S); (Q) Early anther, erect, round, bilobate with longitudinal lines marked on its epidermal tissue (black arrows). (R) Intermediate late stage anther, more quadrate, slightly less erect (apex with some degree of inflection), cucullate anther cap visible. (S) Galeate anther, totally inflected, cucullate anther cap. A pair of pollinia exposed with two laminar caudicles (one pair, white arrows). *Chysis laevis* (T, U, V); (T) Early erect anther, apex pronounced (trapezoid shaped), half round shaped, bilobate with longitudinal lines marked on its epidermal tissue (black arrows). Two very inconspicuous rostellum primordia (white arrows). (U) Intermediate late stage anther, slightly less erect (apex with some degree of inflection), bilobate, anther cap visible. Rostellum appendages vestiges underdeveloped (white arrows). (V) Incumbent anther, the anther cap conical shaped (like a hood), coalescent (fused by the sides to the column), dehiscent pollinia, open, without caudicles. No rostellum developed, only two appendixes underdeveloped and rolled on themselves (white arrows). *Isochilus major* (W, X, Y); (W) Early erect anther, semi-rectangular vertical with a pointed apex, biloculate. Column narrow and slender. (X) Intermediate late stage anther, semi rectangular shaped, pointed apex of conspicuous thickened cells, biloculate, each locule with longitudinal division (black arrow). (Y) Four elliptical inflected pollinia. Anther cap was not observed in this stage because it was lost during dissection; the lone structure (anther cap) was placed and observed at the side of the column in the slide (white arrows). *Ponera juncifolia* (Z, A′, B′); (Z) Early erect anther, rounded and bilobed, each lobule ovoid (white arrows). (A′) Intermediate late stage anther with caudicles exposed. (B′) Anther cap very conspicuous with actinocytic stomata embedded (black arrows). Two pollinia visible in each locule, four long caudicles exposed. *Stelis ciliaris* (C′, D′, E′) (C′) Early anther erect bilobed, each lobule ovoid with a small rounded apex, anther cap beginning its differentiation from the tissue of the rest of the anther, a clearly marked line delimits it (black arrows). (D′) Intermediate late stage anther, bilobed, anther cap clearly marked (black arrows) below two ovoid pollinia in close contact to upper part of rostellum tip (bifid viscidium, white arrow). (E′) Incumbent anther, anther cap with inverted heart shape, two pollinia exposed, in close contact to the tip of rostellum the bifid viscidium with abundant secretions. *Specklinia digitale* (F′, G′, H′) (F′) Inflected anther, cucullate, tip of two pollinia exposed. Column beneath with two teeth (white arrows). Below column narrows and canaliculated (black arrows). Column wings differentiated. (G′) Anther slightly more inflected, column with two teeth (white arrows). (H′) Inflected cucullate anther, two caudicles exposed, column is slim and long, canaliculated in the middle portion (black arrows), and with vertical wings. *Laelia speciosa* (I′, J′, K′) (I′) Erect anther, quadrate, bilobed, with a trapezoidal apex. Lobules with median longitudinal division each (black arrows). Column short and narrow. (J′) Intermediate late stage inflected anther, anther cap sub-globose with round striated cells. Underneath four caudicles visible. (K′) Inflected anther, the anther cap is oblong, with its apex folded up. Four large granulose caudicles are exposed in two pairs. *Oestlundia ligulata* (L′, M′, N′) (L′) Early erect anther, ovoid, bilobed, upper part by the apex a pleat of tissue is formed, early differentiation of the mid-part of the anther cap. (M′) Intermediate late stage inflected anther, bilobed with anther cucullate. (N′) Inflected anther, sub-cubic almost straight, two pollinia visible. *Prosthechea squalida* (O′, P′, Q′); (O′) Early erect anther, oblong and bilobed. (P′) Intermediate late stage inflected anther inflected the anther cap ellipsoid. Under anther cap four pollinia, two pairs of caudicles exposed (white arrows). (Q′) Inflected anther, anther cap truncate obovate shaped. Under it four pollinia, with four large, granulose caudicles in two pairs. *Encyclia microbulbon* (R′, S′, T′). (R′) Inflected anther quadrate with upper corners rounded, under anther cap two pollinia can be seen, (S′) More inflected anther, sub-quadrate with upper part bi-lobed and corners rounded (white arrows). At the apex of anther cap tip of two pollinia exposed. (T′) Inflected anther, obcordate semi-quadrate. Anther cap with abundant semi-actinocytic stomata (black arrows). Below anther cap, four pollinia are visible and two pairs of caudicles exposed. Scale Bars: A, B, C, D, E, F, G, H, J, K, L, M, W, X, Y, Z, A′, B′, C′, D′, E′, F′, G′, H′, L′, M′, N′, O′, P′, Q′, R′, S′ = 100 µm. I, N, O, P, Q, R, S, T, U, V, I′, J′, K′ = 1 mm. Abbreviations: *, anther apex; a, anther; ac, anther cap; ap, appendage; au, auricle; c, column; cau, caudicle; cl, clinandrium; dp, dehiscent pollinia; h, hamulus; l, lip; lo, lobule; lp, lateral petal; m, mentum; o, ovary; p, pollinium; r, rostellum; s, stomata; st, stigma; t, tegula; v, viscidium; w, column wings. Photo Credit SEM Images: Silvia Espinosa Matías. Figure edition: Benjamín Valencia-Nieto.

**Figure 3:**
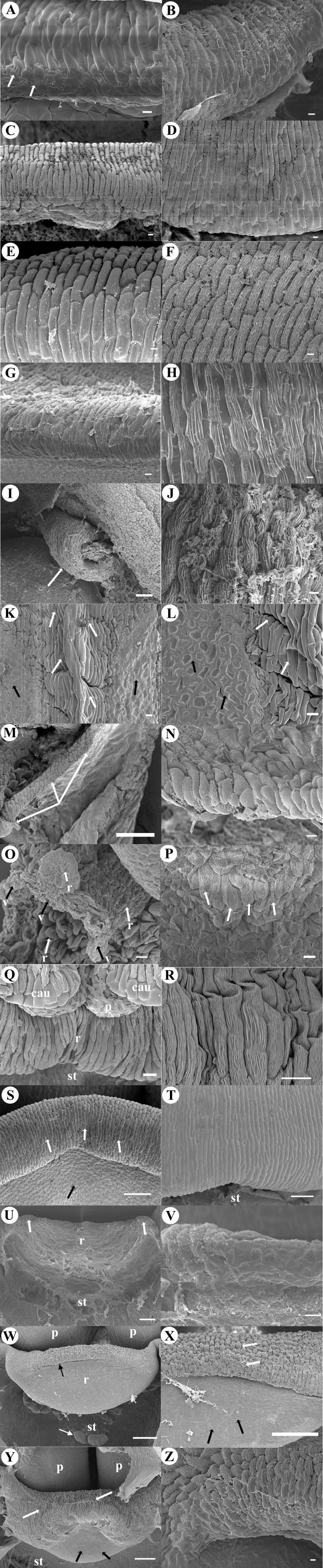
Rostellum micrographs. Rostellum development of *Coelia triptera* (A, B).**** (A) Early stages, smooth rectangular cells, numerous intercellular connections (white arrows). (B) Rostellum at anthesis, wide barrier of rectangular cells. *Bletia purpurea* (C, D). (C) Early incipient rostellum. (D) Rostellum at anthesis (half-moon shape structure) of rectangular epidermal cells. *Chysis limminghei* (E, F). (E) Tip of early-rostellum with rectangular epidermal cells with striate cuticle. (F) Rostellum shield-like made up with rectangular epidermal cells arranged in palisade. *Chysis bractescens* (G, H). (G) Tip of projecting early-rostellum with rectangular epidermal cells. (H) Rostellum shield-like (rectangular epidermal cells in palisade). *Chysis laevis* (I, J). (I) Close-up of one of two appendixes of underdeveloped rostellum rolled on itself (white arrow). (J) Underdeveloped rostellum made up with striated rectangular epidermal cells arranged in palisade. *Isochilus major* (K, L). (K) Triangular rostellum with two cellular types: elongated cells (white arrows), and the glandular cellular type (black arrows) (L) Close-up of two cellular types of rostellum, the viscidium (black arrows). *Ponera juncifolia* (M, N). (M) Right side of v-shaped shield rostellum (white arrows) (N) Closer view of the border of rostellum with fibrous cells. *Stelis ciliaris* (O, P). (O) Tip of upper part of rostellum, the viscidium is bifid with very fibrous cells, in the middle the tissue is covered with a dense secretions (black arrows) (P) Rostellum with big round fibrous cells (white arrows). *Specklinia digitale* (Q, R). (Q) Rostellum with large longitudinal striated cells. (R) Close-up of rostellum. *Laelia speciosa* (S, T), (S) Tip of early rostellum with semi-rectangular cells (white arrows), the abaxial side with smooth irregular cells (black arrow). (T) Close-up of rostellum at anthesis. *Oestlundia ligulata* (U, V); (U) Transverse half-moon shaped rostellum. (V) Rostellum tip. *Prosthechea squalida* (W, X). (W) Early ellipsoid rostellum, border with thickened cells (black arrow two small bulges of stigma (white arrow). (X) Close-up of the superior part or rostellum (white arrows), lower part with smooth cells (black arrows). *Encyclia microbulbon* (Y, Z) (Y) Early pointy tongue-shaped rostellum with longitudinal elongated striated cells (white arrows), the abaxial side is smooth (black arrows). (V) Close-up of the striated cells at the tip of rostellum. Scale Bars: A, B, C, D, E, F, G, H, J, K, L, N, O, P, Q, R, S, T, *Z* = 10 µm. I, M, U, V, W, X, *Y* = 100 µm. Abbreviations: cau, caudicle; p, pollinium; r, rostellum; st, stigma; v, viscidium. Photo Credit SEM Images: Silvia Espinosa Matías. Figure edition: Benjamín Valencia-Nieto.

**Figure 4:**
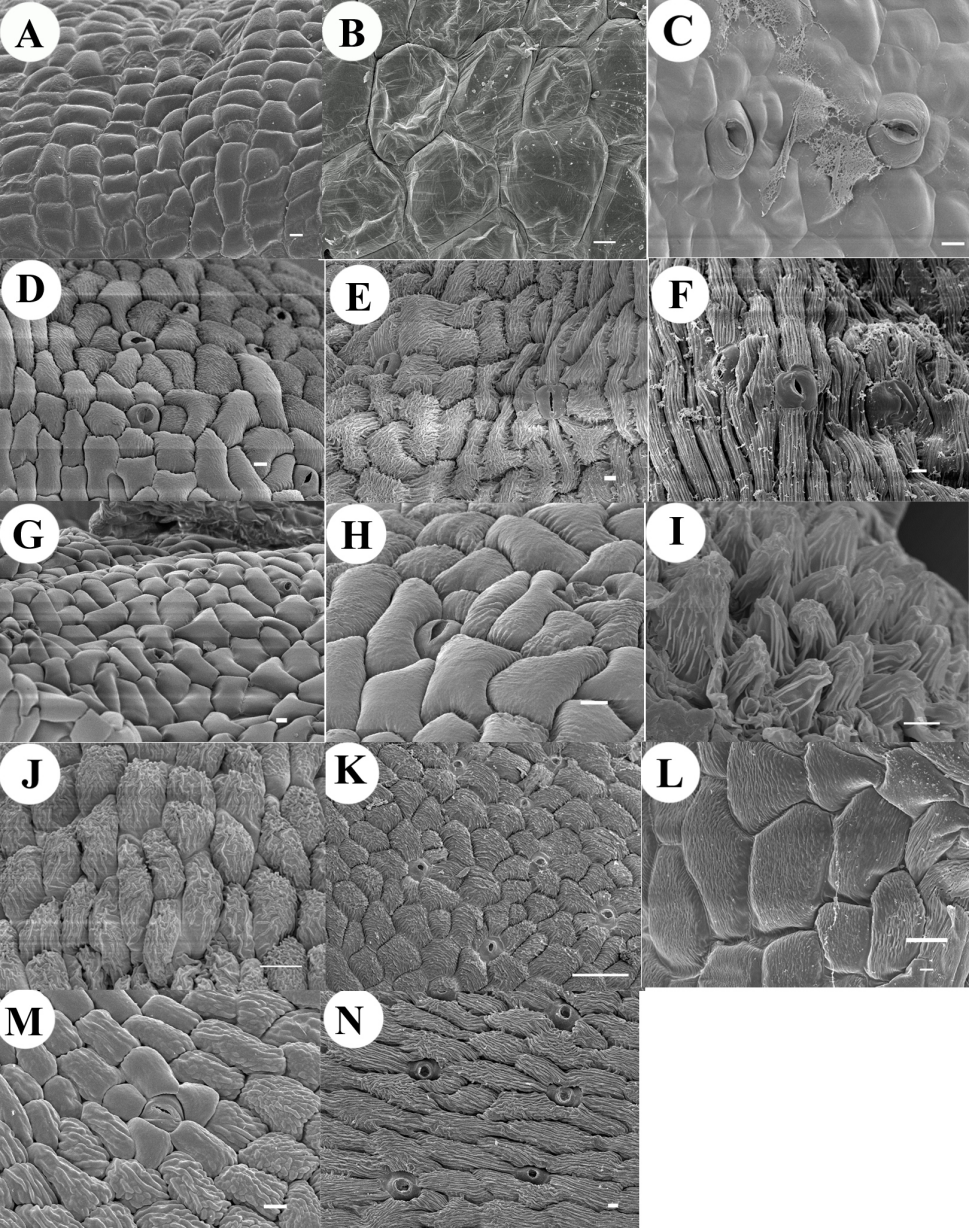
Micrographs of anther cap cuticle. Cuticle of the epidermis of the anther cap of (A) *Corallorhiza maculata* with smooth cells without stomata. (B) *Govenia alba* with smooth to slightly wrinkled cells** without stomata. (C) *Coelia triptera* with smooth cells showing actinocytic stomata and secretions. (D) *Bletia purpurea* with slightly striated cells, note the actinocytic stomata. (E) *Chysis limminghei* with conspicuous striated cells and actinocytic stomata. (F) *Chysis bractescens* with longitudinal striated cells and actinocytic stomata. (G) *Isochilus major* with smooth cells and actinocytic stomata. (H) *Ponera juncifolia* with smooth to slightly striated cells and actinocytic stomata. (I) *Stelis ciliaris* with striated slightly corrugated cells without stomata. (J) *Specklinia digitale* with densely striated cells without stomata. (K) *Laelia speciose* with round striated cells and actinocytic stomata. (L) *Oestlundia ligulata* with round slightly striated cells without stomata. (M) * Prosthechea squalida* with slightly wide striated cells and actinocytic stomata. (N) *Encyclia microbulbon* with conspicuous striated cells and semi-actinocytic stomata. Scale Bars: A–J, L–N = 10 µm, *K* = 100 µm. Photo Credit SEM Images: Silvia Espinosa Matías. Figure edition: Benjamín Valencia-Nieto.

**Figure 5:**
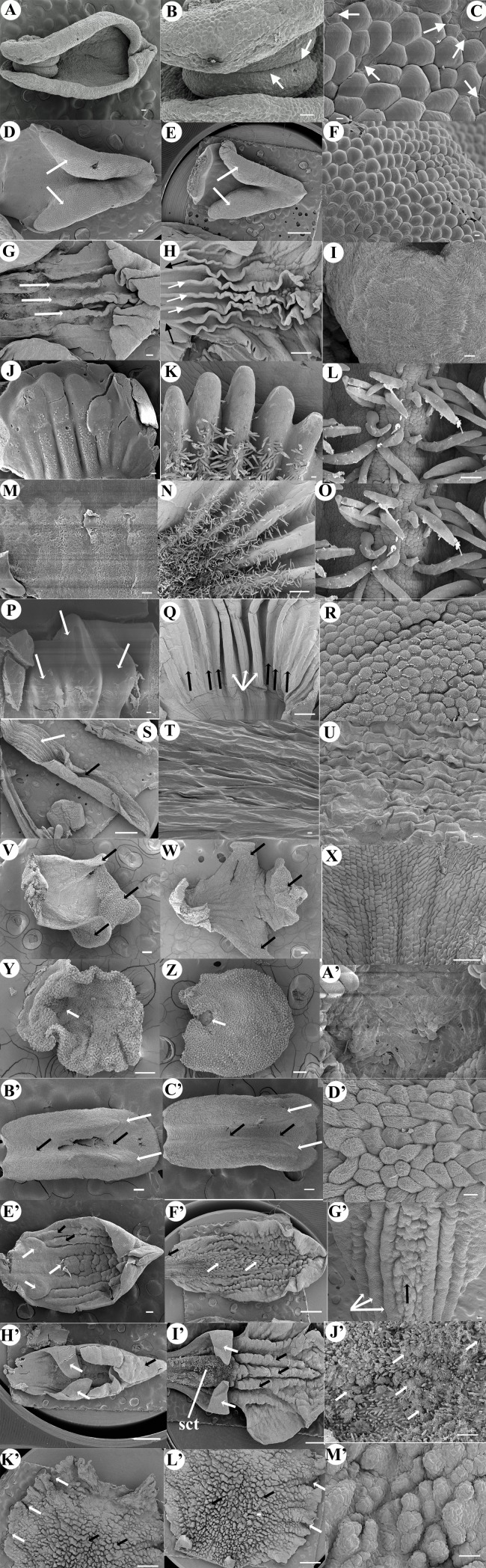
Early and anthesis stages of lip development. * Corallorhiza maculata* (A, B, C). (A) Lip. (B) Protuberances of lip forming a channel in the middle. (C) Close-up of the cells of the protuberances, presence of abundant pores. *Coelia triptera* (D, E, F). (D) Early lip development with two bulges (white arrows). (E) Lip at anthesis arrowhead shaped bulging sides (white arrows). (F) Smooth rounded single epidermis cells. *Bletia purpurea* (G, H, I). (G) Early stage lip (three central keels white arrows). (H) Anthesis lip (three central keels with white arrows and 3–4 lateral accessory keels developed with black arrows). (I) Epidermis cells. *Chysis limminghei* (J, K, L). (J) Early lip development, five central keels with large papillae epidermal cells protruding. (K) Lip at anthesis, five keels with abundant simple trichomes. (L) Close-up of trichomes. *Chysis bractescens* (M, N, O). (M) Early lip development, five central keels with large papillae epidermal cells protruding. (N) Lip at anthesis, five keels with abundant large simple trichomes. (O) Close-up of trichomes. *Chysis laevis* (P, Q, R). (P) Early lip development with three central keels prominent (white arrows). (Q) Base of the lip at anthesis, three central keels (white arrows), five accessory keels at each side of the lip (black arrows). (R) Close-up of epidermal cells of the keels. *Isochilus major* (S, T, U). (S) Lip at anthesis, flat channeled (white arrow), forming a slender channel (black arrow). (T) Close-up of epidermal cells of the flat part of lip. (U) Close-up of epidermis cells of the conduplicate part and lip apex. *Ponera juncifolia* (V, W, X). (V) Early lip development, tri-lobed (black arrows). (W) Lip at anthesis, mid-lobe apex slightly wrapped (black arrows). (X) Epidermis cells. *Stelis ciliaris* (Y, Z, A′). (Y) Early lip, at base hollow in the middle (white arrow). (Z) Lip at anthesis, hollow at base (white arrow) with trichomes. (A′) Close-up of hollow with trichomes. *Specklinia digitale* (B′, C′, D′). (B′) Early lip, two simple thickened keels (white arrow), a channel formed between them (black arrows). (C′) Lip at anthesis with two simple thickened keels (white arrows) and channel between (black arrows). (D′) Cells on the keels. *Oestlundia ligulata* (E′, F′, G′). (E′) Early lip with three central keels (white arrows), two accessory keels at each side (black arrows). (F′) Lip at anthesis unguiculate to the base (black arrow), central keels highly verrucose (white arrows). (G′) Central highly verrucose keel (white arrow) besides accessory keels black arrows). *Prosthechea squalida* (H′, I′, J′). (H′) Tri-lobed lip, the tip wrapped on itself (black arrow), two small lateral lobes are incurved (white arrows). (I′) Unguiculate lip at the base, tri-lobed, the lateral lobes incurved (white arrows), the central portion is composed of abundant glandular trichomes. From the center to the tip of the mid-lobe, three large central verrucose keels (black arrows) and two or more lateral keels are present. (J′) Central**** portion with abundant glandular trichomes secreting large amounts of sugar crystals (white arrows). *Encyclia microbulbon* (K′. L′. M′). (K′–L′) Mid-lobe lip, circular-ovate with crenulated margins (white arrows), the center the surface is very verrucose (black arrows) without keels. (M′) Close up of verrucose surface cells. Scale Bars: C, F, I, R, T, U, A′ = 10 µm, A, B, D, G, J, K, L, M, O, P, V, W, X, Y, Z, B′, C′, D′, E′, G′, J′, M′ =100 µm. E, H, N, Q, S, F′, H′, I′, K′, L′ = 1 mm. Photo Credit SEM Images: Silvia Espinosa Matías. Figure edition: Benjamín Valencia-Nieto.

**Figure 6:**
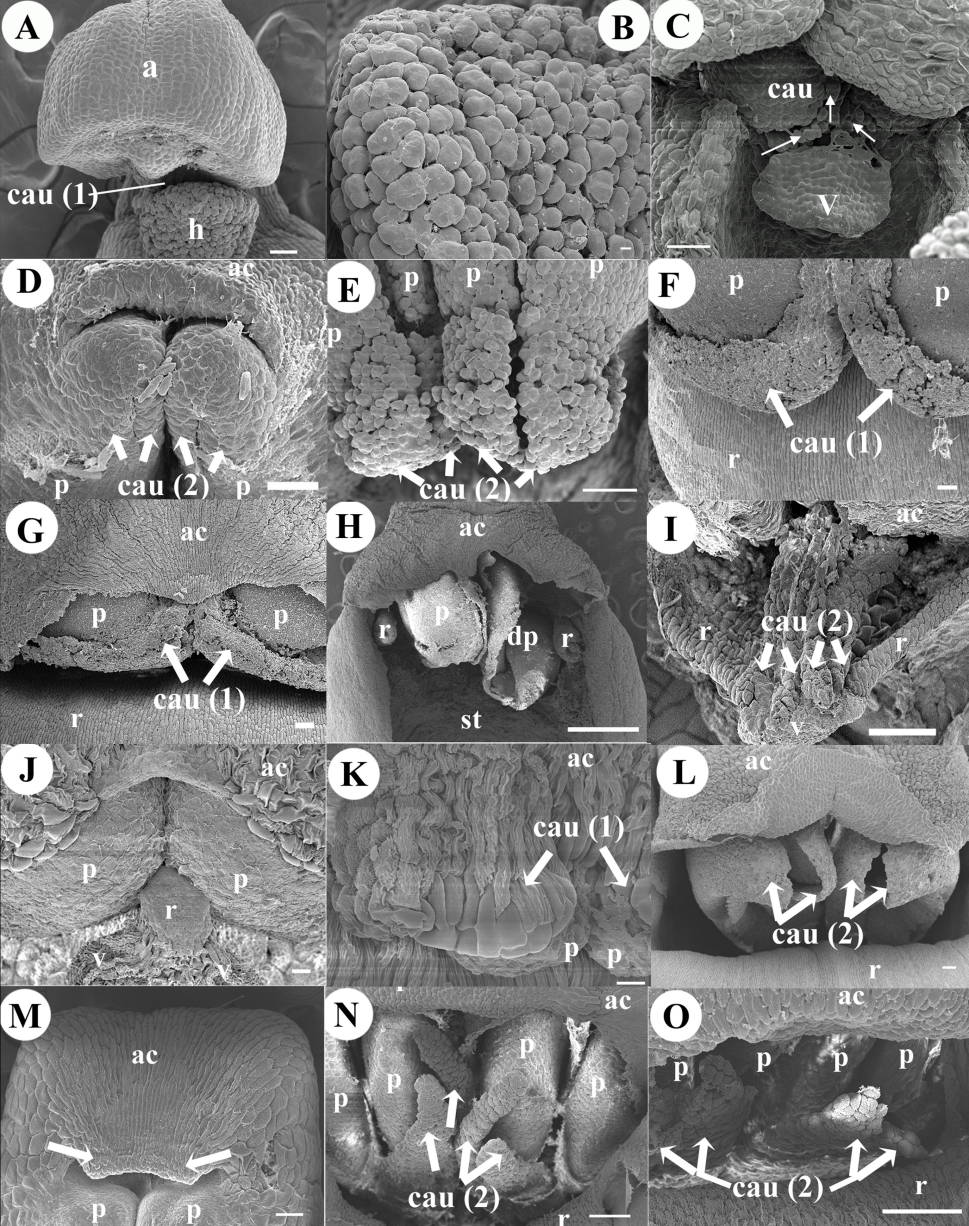
Micrographs of pollinia and caudicles. Pollinia and caudicles of (A) *Corallorhiza maculata* one caudicle connected to hamulus. (B) *Corallorhiza maculata* smooth, very rounded, granulose cells of the hamulus. (C) *Govenia alba* **** tegula composed by a strand of granulose cells with viscidium resembling a little shield, connected to the apex of the pollinia (one pair). (D) *Coelia triptera*,** two pollinia and two pairs of caudicles with polyhedral cells. (E) *Bletia purpurea*,** four upper pollinia and two pairs of caudicles with polyhedral cells. (F) *Chysis bractescens*,** two pollinia and one pair of linear caudicles with membranaceous cells. (G) *Chysis limminghei*,** two pollinia and one pair of linear caudicles with membranaceous cells. (H) *Chysis laevis*, two pollinia, one pollinium without caudicles and another dehiscent** pollinium. (I) *Ponera juncifolia*, note two pairs of caudicles, pollinia covered with anther cap. (J) *Stelis ciliaris,* two pollinia and central bifid tip of rostellum. (K) *Specklinia digitale*, two pollinia and one pair of rectangular smooth caudicles. (L) *Laelia speciosa* with four large granulose caudicles exposed in two pairs. (M) *Oestlundia ligulata*, two pollinia and pleat of tissue in the apex (white arrows) of the anther cap. (N) *Prosthechea squalida*, four pollinia and two pairs of caudicles. (O) *Encyclia microbulbon*,** four pollinia and two pairs of caudicles. Scale Bars: A, C,**** D, E, F, G, I, M, N, L**** = 100 µm. B, J, *K* = 10 µm. H =1 mm. Abbreviations: ac, anther cap; cau, caudicle (irregular mass of cells); cau (1), one pair of caudicles; cau (2), two pair of caudicles; h, hamulus; p, pollinium; r, rostellum; st, stigma; v, viscidium. Photo Credit SEM Images: Silvia Espinosa Matías. Figure edition: Benjamín Valencia-Nieto.

***Early late stages*: Anther** is inflected (early incumbency), about 90° to the axis of the column, epidermis is smooth without ornamentation. At its apex, one **caudicle** is in close contact with the apical part of the rostellum, which in this portion is composed of viscidium cells. The narrowed apex of the **column** is slightly bent over itself, the column widens toward the base, the auricle (a small protuberance) is present at the sides (Fig. 2A). ***Anthesis:*** **Anther** has grown, remaining sub-globose, operculate shaped and incumbent; it looks as if has experienced marked inflection, but this is the result of the pronounced bending over itself of the apical portion of the column (Figs. 2B–2C). From the middle to the apex, the **column** is pronouncedly bent over itself and it appears as if it were shorter in its length than in the previous developmental stage (Fig. 2C). The column widens towards its base, where the two auricles are located, these auricles are prominent, thick and extending along the sides, underneath where the column starts; the ovary has the same width (Figs. 2B–2C). We have not documented the rostellum development in this taxon, the Figs. 3A–3B contains *Coelia triptera* rostellum development as only representative of this subtribe. A well differentiated **anther cap** is absent; the cells of the epidermis of the anther are smooth to slightly striated (Fig. 4A). Edges and apex of **lip** are incurved; two lamellae (protuberances) are present at the base of the lip, these are longer than wide, forming a channel in the middle; between the borders where these two protuberances are in contact, in a close-up of the cells of this zone, the presence of abundant pores are observed as in the initial stages (Figs. 5A–5C). Below the apex of the anther, the **hamulus** protrudes in the center as a very conspicuous structure. Behind it, the rest of the rostellum is extending under the basal part of the anther (Fig. 6A). The cells of the hamulus in close-up are smooth, rounded, granulose (Fig. 6B). The apex of the **lip** and its edges are extended.

### Subtribe Calypsoinae: *Govenia alba*

***Early late stages:*** the incumbent **anther** is approximately 90° from the axis of the column (early incumbency), is unilobed and sub-quadrate shaped. Epidermal cells are smooth to slightly wrinkled. In the middle, below the apex of the anther, a prominent sub-oval viscidium is visible, connected by means of an acute stipe (tegula). The **column** is erect and about the same width along its entire length. Small wings begin to develop along its sides (Fig. 2D). We have not documented the rostellum development in this taxon, the Figs. 3A–3B contains *Coelia triptera* rostellum development as only representative of this subtribe*.* ***Anthesis:*** **Anther** is inflected approximately 170° to the axis of the column, and grown noticeably with an ovoid shape; in the middle part, it presents a very prominent keel made of uneven conspicuous cells (Figs. 2E–2F); the rest of the epidermis of the anther is made of smooth to slightly wrinkled cells (Figs. 2F and 4B). The **column** is of the same width along its length and pronouncedly bent over itself; on its sides, the broadly rounded, oblong wings are slightly unfolded (Fig. 2E). We have not documented the lip development in this taxon, the Figs. 5A–5C contains *Corallorhiza maculata* as only representative of this subtribe. In the middle of column, underneath the apex of the anther, a peltate viscidium is present, and a **tegula** composed of a strand of granulose cells visible (Fig. 6C).

### Subtribe Calyosoinae: *Coelia triptera*

***Early late stages:*** **Anther** is inflected approximately 35° to the axis of the column (early incumbency), bilobed, and the tip of the anther widens toward the apex; just below the anther apex, two small rounded protuberances are visible; the **anther cap** is not distinguishable from rest of the anther epidermis. The **column** is hexagonal (Fig. 2G). ***In the intermediate late stage:*** the **anther** is slightly more inflected, about 45° to the axis of the column, remains bilobed with a conspicuous apex, and the two small rounded protuberances are more developed. The cuticle of the epidermis of the anther is composed mainly of rounded, smooth cells, but the **anther cap** is still not distinguishable (Fig. 2H). At the base of the anther, the developing rostellum consists of a transverse structure with smooth rectangular cells, in the lower layer of this tissue numerous intercellular connections are observed between the walls (Figs. 2H and 3A). No conspicuous clinandrium is observed, the **column** is short. ***Anthesis:*** **Anther** is totally inflected; its apex is now located approximately 90° from its original position (the axis of the column. Figs. 2I, 2J). The **column** is hexagonal; at its base it joins to the ovary which has pointed projections; it is very wide, with large longitudinal grooves (Fig. 2I). The well-developed rostellum forms a morphological barrier to separate the fertile stigmatic surface, which is basal (similar to the aperture of a cavern. Figs. 2I, 2J and 3B). The **anther cap** is now clearly distinguishable, shows smooth cuticular rounded cells with few smooth actinocytic stomata with secretions (Fig. 4C). In the initial stages the **lip** is simple, arrowhead shaped without ornamentations or projections on the adaxial face, only with bulging sides like anticline convex folds, converging in the apex (Fig. 5D). By anthesis the **lip** has grown in length and conserves the attributes previously described; and in close-up its cells are rounded and smooth (Figs. 5E–5F). Beneath the anther cap, on the tip of each pollinium, are two pairs of round **caudicles** with polyhedral cells (according to the previous description of Salazar (1990); they are granulose. Figs. 2J,  6D).

### Subtribe Bletiinae: *Bletia purpurea*

***Early late stages:*** **Anther** begins its differentiation as an erect structure, it is subquadrate, bilobed, with trapezoidal apex distally. Below the base of the anther, the **column** shows two very small lateral appendages that correspond to initial clinandrium phases (Fig. 2K), on the middle, a transverse ridge of tissue is beginning its development, corresponding to the initial stages of the rostellum (Fig. 3C); below the proto-rostellum there is a semicircular area composed of granulose tissue, corresponding to the incipient surface of the stigmatic tissue (Fig. 2K). ***In the intermediate late stage:*** the **anther** is round (spherical in three-dimensional view); it is inflected, the apex is located approximately 160° from its original position, the cucullate **anther cap** is now visible, with rounded epidermal cells and abundant actinocytic stomata on its upper part; below the cells are less conspicuous, smaller and rectangular. On the underside of the anther cap, the two lobes show signs of a longitudinal division. At this stage, the **column** is more developed and broadened. The two lateral appendages observed at the column apex sides are now broadened, clearly forming the clinandrium; by the center, the transverse ridge of tissue forms a defined rostellum, now projected as a well-developed tongue shaped structure with rectangular epidermal cells. Underneath is the stigmatic surface, covered by the well-developed rostellum (Fig. 2L). ***Anthesis:*** the 170° inflected **anther** looks obtriangular, with rounded edges; a fully developed **anther cap** is composed of an epidermis with irregular striated cells, the actinocytic stomata are less visible but present in the same area of previous stage. (Fig. 2M and 4C). Underneath the anther cap, fully developed pollinia are exposed, the four upper ones are visible and beneath them are located another four (as previous reported in the genus by Sosa, 2002 and Sosa, 2008); the polyhedral cells developed on the apex of each pollinium are more rounded and thickened, forming the **caudicles** (Figs. 2M and 6E). The anther is seated in the well-defined clinandrium, the rostellum has a semi-lunate shape, forming a shield separating the pollinarium from the fertile surface of the stigma located below (Figs. 2M and 3D). The sides of the **column** are spreading, forming the column wings (Fig. 2M). At initial stages on the **lip**, three central keels can be observed extending longitudinally from the base to the apex (Fig. 5G). By anthesis, three central and few accessory keels can be observed extending longitudinally from the base to the apex. (Fig. 5H). The cells of the epidermis of the lip are striated (Fig. 5I).

### Subtribe Bletiinae: *Chysis bractescens*, *Chysis limminghei* and *Chysis laevis*

***Early late stages:*** the **anthers** of the three species begin their differentiation as erect structures, in *C. bractescens* and *C. limminghei* are round and in C. *laevis* more semi-circular or half round, all are bilobed, with each lobule presenting a longitudinal line marked on the epidermal tissue (Figs. 2N, 2Q, 2T); *C. laevis* shows a tissue in its apex (trapezoid shaped) and in the other two species the apex is not pronounced. At the base of the anther, towards the front, the column of *C. bractescens* and *C. limminghei*, respectively, form an early developing rostellum, projecting conspicuously upward like a semi-lunate shape, covering about half of its length (Figs. 2N,  2Q and 3E, 3G). However, at the same stage of C. *laevis* only two very inconspicuous projections or rostellum primordia can be observed below the anther (Fig. 2T). No development can be seen of clinandrium appendices in any of the three species; nor stigmatic surface or receptive tissue. In the middle of the **lip** of *C. bractescens* and *C. limminghei* five keels are beginning to develop; some epidermal papillae cells (look bigger and rounded) begin to differentiate and protrude in the middle of each keel (Figs. 5J, 5M). In *C. laevis*, only three wide keels are developing, without different cells protruding on its epidermis (Fig. 5P). ***In the intermediate late stage:*** the **anther** form on *C. bractescens* and *C. limminghei* is more quadrate, slightly less erect, its upper part above the two lobules now has a more defined anther cap (Figs. 2O, 2R). The rostellum is projecting towards the front in *C. bractescens* and *C. limminghei*, but in *C. laevis* the rostellum appendages remains underdeveloped and are only slightly more pointed (Fig. 2U). On *C. limminghei* and *C. laevis*, a transversal cleavage of the tissue has formed; some secretions can be seen in this area, that correspond to the future receptive stigmatic surface. The **column** apex of *C. bractescens* and *C. limminghei* has widened and two small, lateral clinandrium teeth are observed (Figs. 2O, 2R); similarly, the column is widened also in *C. laevis*, but clinandrium appendices or teeth are no present (Fig. 2U). ***Anthesis:*** the fully developed **anthers** of the three species are inflected, their apex is now located about 170° from its original position (Figs. 2P, 2S, 2V). In *C. bractescens* and *C. limminghei* is galeate shaped, in both, the cucullate **anther cap** (shaped like a helmet with mask) covers almost completely the pollinia, and its epidermal cells have irregular striated cuticle with few actinocytic stomata (Figs. 4E, 4F). Under the anther cap, in the apex of the anther a pair of pollinia is exposed, bearing two laminar **caudicles** composed of a membranaceous zone (as observed by Soto-Arenas, 2008a in fresh flowers. Figs. 6F, 6G). Below, the transverse rostellum forms a very conspicuous structure, like a rectangular shield, made up with epidermal cells of rectangular palisade shape (Figs. 2P, 2S and 3F, 3H). Underneath, the cleavage has widened and is covered with a thick layer of secretions. The **column** is very wide, laterally two teeth, one at each side of the column, are observed. Differently, in *C. laevis* the cover of the **anther** is conical, like a hood, coalescent apparently fused by the sides to the **column**; underneath, the pollinia are dehiscent, open, without caudicles (Figs. 2V and 6H); there is no rostellum developed, only two appendixes, underdeveloped, rolled on themselves are seen at the sides of the column (Figs. 2V and 3I, 3J). In the middle part of that zone there is a large hollow with secretions on its surface, corresponding to the receptive area of the stigma. The column is elongated and not very wide. The five keels on the **lip** of *C. bractescens* and *C. limminghei* are fully developed, numerous simple trichomes and papillae are present along the middle part of each keel (Figs. 5K, 5L, 5N, 5O); distinctively, in *C. laevis* only three large keels are fully developed and several less conspicuous accessory keels have arisen, four on each side of the lip, and trichomes are not present (Figs. 5Q, 5R).

### Subtribe Ponerinae: *Isochilus major*

***Early late stages:*** the **anther** begins its differentiation erect, elongate, bilobed, semi-rectangular vertical, with a pointed apex composed of large conspicuous cells; at the base of the anther at the front the early developing rostellum is deltoid, projecting conspicuously upward. The **column** is narrow and slender (Fig. 2W). ***In the intermediate late stage:*** the **anther** has inflected around 45° from its original position, continues semi-rectangular shaped, bilobed, with pointed apex of conspicuous large cells; each lobule has now a line in the middle part (an indication of its undergoing a longitudinal division, Fig. 2X); observations of the **anther cap** epidermis reveals the presence of actinocytic stomata embedded in the smooth epidermal cells (Fig. 4G). On the middle part, in front of the base of the anther, the acute rostellum is now projecting towards the front, it is composed of two cellular types: one, made of longitudinal elongated cells; and at the two sides of the central part, the other cellular type corresponds to glandular tissue made of rounded cells and copious secretions throughout its diameter, which corresponds to the viscidium (Figs. 3K, 3L). At each side of the column, prominent lateral teeth are observed forming the clinandrium. The **column** is very narrow, elongated (Fig. 2X). ***Anthesis:*** The **anther** is completely inflected around 180° from its original position, has four elliptical pollinia exposed: the **anther cap** was not observed (because it was lost when dissections were made). Clinandrium is three-toothed, two are lateral teeth and one prominent central tooth. The **column** continues narrow and more elongated (Fig. 2Y). The **lip** is long and narrow, at its base is flat channeled, near the median part its very folded over, forming a slender channel extending to the entrance formed by the acute apex, two different cellular types are present on these zones of the epidermis (Figs. 5S–5U).

### Subtribe Ponerinae: *Ponera juncifolia*

***Early late stages:*** The **anther** initiates erect, rounded and bilobed, each lobule is ovoid (Fig. 2Z); the cuticle of the epidermis cells of the **anther cap** is almost smooth, with actinocytic stomata embedded along the upper part (Fig. 4H). At the base of the anther, the obtriangular rostellum is projected to the front, and two cellular types are distinguishable on it: those extending from the base to the middle part of the inverted triangle which are slender longitudinal elongated cells; in the apex, the other cellular type is composed of glandular tissue with rounded cells with copious secretions throughout its diameter (as described above in *I. major*) which corresponds to the viscidium (Figs. 2Z, and 6I). Basally, the **column** has a “v” form without visible clinandrium. The **lip** is tri-lobed, without keels or ornamentations; the mid-lobe tip is very folded over upon itself (Fig. 5V). ***Anthesis:*** the **anther** has inflected about 90° from its original position; the **anther cap** is very conspicuous, with an epidermis of some large smooth cells, and other slightly striated cells and actinocytic stomata embedded (Figs. 2A^′^, 2B^′^ and 4H). In the median zone by the apex, where the anther cap is not obstructing view, two pollinia are visible in each lobule, and four long caudicles are exposed, the tip of those **caudicles** are closely in contact with the viscidium (Figs. 2B^′^ and 6I). The rest of the rostellum forms a well-defined v-shaped shield with the border made of rectangular cells (Figs. 2B^′^ and 3M–3N). Two small protuberances are seen at the sides of the column forming a small clinandrium (Fig. 2B^′^). Tri-lobed **lip** tips are extended and the cells of its middle part are sub-quadrate shaped and striated (Figs. 5X–5Y).

### Subtribe Pleurothallidinae: *Stelis ciliaris*

***Early late stages:*** The **anther** is erect, bilobed, each lobule is ovoid with a small rounded apex, the upper part of the **anther cap** is beginning its differentiation, its tissue is clearly marked with a line that delimits the two epidermal cell types (Fig. 2C^′^). The epidermis of the anther cap appears corrugated. At the base of the anther, hanging at the center in the mid part on the tip of rostellum, the viscidium forms one bifid appendage, the cells in the tip of this appendage are very conspicuous, and the cells of its exterior border are very conspicuously rounded. Below this area there is a hollowed structure (Fig. 2C^′^). The cordate **lip** is not well extended and has crenulated margins, its epidermis has very conspicuous rounded cells, at the base in the center has a hollow structure known as glenion (Fig. 5Y). ***In the intermediate late stage:*** the **anther** is inflected about 90° from the original position, bilobed, and the **anther cap** has big striated epidermal cells (Figs. 2D^′^ and 4I). Below the anther cap, two ovoid pollinia are in close contact with the upper part of the bifid rostellum tip (the viscidium), the rest of rostellum has large round cells (Fig. 2D^′^). ***Anthesis:*** the **anther** has inflected approximately 170° from its original position, the **anther cap** has and inverted heart shape (Fig. 2E^′^), its epidermis with conspicuous, collapsed, corrugated cells (Fig. 4I). In the center by the apex of the anther, below the anther cap two pollinia are exposed, these two pollinia are in close contact to the upper rostellum and on its tip, whose sides are covered with a dense secretion corresponding to the viscidium (Figs. 3O and 6J). The rest of rostellum has big round cells (Figs. 2E^′^ and 3P). The stigma is prominent, standing out as the ends of an olive wreath at each side of the anther (Fig. 2E^′^). **Lip** is extended cordate, in the base the glenion is full of trichomes embedded with secretions (Figs. 5Z, 5A^′^).

### Subtribe Pleurothallidinae: *Specklinia digitale*

Only the intermediate late stages and anthesis stage of this species were observed. ***In the intermediate late stage:*** the apex of the **anther** is 145° from the axis of the column, its form is cucullate, the tips of two pollinia are exposed. Beneath, the crenulated rostellum forms a transverse morphological barrier made of large longitudinal cells. Just under it, there is a hole, the **column** beneath this entrance has two teeth. Below, the column narrows and at center is canaliculate and the clinandrium is a small lobe behind the anther. The column wings are differentiated on the top and their tips are like teeth at each side (Fig. 2F^′^). In a subsequent stage the anther is slightly more inflected than in the previous stage (near 155°). Rostellum is narrower and not crenulated anymore, forming a transverse morphological barrier made of large longitudinal cells. Just under it, there is a depression where the stigma is located; the column beneath this entrance has two teeth. Below, the column elongates, narrows and is canaliculate. The column wings are slightly more differentiated and widespread (Fig. 2G^′^). The **lip** is simple, narrow, ligulate, slightly arched with two simple thickened keels, extending from the base nearly to the apex (Fig. 5B^′^). ***Anthesis***: The **anther** is cucullate (Fig. 2H^′^), two pollinia with the **caudicles** exposed, the cells on its tips are large and smooth, claw shaped (Fig. 6K). The anther apex is 170° from the axis of the column. In this stage, the epidermis of the **anther cap** has densely striated cells (Fig. 4J). Beneath, the ventral rostellum is forming a transverse morphological barrier made of large longitudinal cells. Just under it there is a hollow with the stigma (Figs. 3Q, 3R). The **column** is slender and long, canaliculate in the middle portion, and with vertical wings; the clinandrium is extended at the sides and along the border of the anther (Fig. 2H^′^). The **lip** is simple, ligulate, slightly arched, with two simple thickened keels with slightly striated round cells, extending from the base nearly to the apex (Fig. 5C^′^, Fig. 5D^′^).

### Subtribe Laeliinae: *Laelia speciosa*

***Early late stages:*** the **anther** is erect, quadrate, bilobed, with a trapezoid apex. The lobules show signs of undergoing a longitudinal division (a line in the middle part of each lobule is observed). Below, a transverse ridge of tissue forms a defined rostellum projecting upwardly as a well-developed structure with rectangular epidermal cells (Figs. 2I^′^ and 3S). Underneath there is an area composed of different tissue corresponding to the incipient surface of the stigmatic region. The **column** is short and narrow, almost the same size as the anther length (Fig. 2I^′^). ***In the intermediate late stage:*** the **anther** has growth massively, is inflected, its apex is now located approximately 80° from its original position; the **anther cap** is sub-globose, with round striated cells. Beneath the apex of the anther cap are visible four **caudicles**. At the base of the anther, a transverse rostellum projects apically. Underneath is a big conspicuous depression where the future stigmatic surface will develop (Fig. 2J^′^). ***Anthesis***: the **anther** has inflected more, its apex is now located approximately 140° from its original position, and the **anther cap** is oblong, with its apex folded up. The upper part of anther cap has the epidermis with striated cells and abundant actinocytic stomata (Figs. 2K^′^ and 4K). Beneath the apex of the anther cap, there are four large granulose **caudicles** in two pairs (Fig. 6L). Below the base of the anther, the large semi-circular rostellum, with slightly striated large longitudinal cells (Fig. 3T) forms a shield that acts as a morphological barrier isolating the fertile stigmatic surface underneath it. The stigma surface is covered with abundant secretions. The **column** is broad, narrowing towards the anther, and the clinandrium margins are toothed (Fig. 2K^′^).

### Subtribe Laeliinae: *Oestlundia ligulata*

***Early late stages:*** the **anther** is erect, ovoid, and bilobed; by the apex a pleat of tissue is formed, corresponding with the early differentiation of the mid-part of the **anther cap**. The rest of the epidermis of the anther cap is not distinguishable from the anther epidermis (Fig. 2L^′^). At the base of the anther, in front of it, the rostellum projects upward, two crests are present in its superior border near the apex. Below the rostellum, a transversal slit is formed along the **column**. The column has two median lateral projections (like teeth) at its sides forming the clinandrium (Fig. 2L^′^). The **lip** is ornate, two rounded bulges are found at the base, extending along toward the tip forming two central keels; two accessory keels are present, on each side, the tip of the lip is folded upon itself (Fig. 5E^′^). ***In the intermediate late stage:*** the **anther** continues erect and bilobed, its upper part is differentiated clearly forming the **anther cap**, which now can be seen cucullate, beneath the two lobules has its external sides very rounded, with marked wrinkles along its epidermis (Fig. 2M^′^). ***Anthesis:*** the now sub-cubic **anther** continues almost straight (bent less than 40° from its original position. Fig. 2N^′^); the **anther cap** has round, slightly striated cells at the apex (Fig. 4L), and the border possesses rounded cells. Only two pollinia are visible (Fig. 6M). Beneath, in the middle, the rostellum is narrower and has two crests by the center near its border (Figs. 2N^′^ and 3U, 3V), the tissue under it has an abundant secretion of unknown nature; the ventral stigma is slightly cordate, with lateral lobes prominent to the margins (Fig. 3U). Two lateral teeth form the clinandrium. Towards the base the **column** narrows (Fig. 2N^′^). The oblong **lip** is elongated and deeply ornamented, its base is unguiculate; the central keel is highly verrucose, flanked by three lateral keels on each side, extending along its length (Figs. 5F^′^, 5G^′^).

### Subtribe Laeliinae: *Prosthechea squalida*

***Early late stages:*** The erect **anther** is oblong and bilobed, each lobule is ovoid, and no differentiation can be observed on the epidermis of the upper area of the anther (that will develop into the **anther cap** Fig. 2O^′^); in a close-up, small actinocytic stomata are observed and are embedded within the striated epidermis (Fig. 4M); below the apex there is a pleat of tissue (Fig. 2O^′^). In the middle an ellipsoid rostellum is seen forming a barrier, underneath the **column** is depressed, in the deeper part, two small bulges of tissue are present (Fig. 3W), their surface is densely hairy, covered with secretion of unknown nature. At each side of the column a small lateral tooth is forming the clinandrium (Fig. 2O^′^). The **lip** is tri-lobed, the tip of large mid-lobe is incurved, with the apical margins wrapped around each other, and the lateral lobes are incurved. Near the base, below those lateral lobes abundant papillae are observed (Fig. 5H^′^). ***In the intermediate late stage:*** the **anther** has inflected nearly 90° from its original position, in the middle, the ellipsoid **anther cap** is well differentiated; under the anther cap four pollinia are present, underneath two pairs of **caudicles** are exposed (Fig. 2P^′^); above the anther a conspicuous mid-tooth of the clinandrium is seen and at its sides, it is flanked by two lateral teeth. Below the base of the anther, a transverse rostellum is present, under which there is a cordate stigma (Fig. 2P^′^). ***Anthesis:*** behind the **anther**, a prominent mid-tooth of the clinandrium is present and it is flanked by the two lateral teeth of the clinandrium (Fig. 2Q^′^). The **anther cap** is truncate with obovate shape. Under the anther cap, four pollinia are present, with four large granulose **caudicles** exposed in two pairs (Fig. 6N). Below the base of the anther, a transverse, brick shaped rostellum is present (Figs. 2Q^′^ and 3X). Below the rostellum, the stigma with abundant stigmatic fluids is observed. The **lip** is unguiculate and tri-lobed. The lateral lobes are small and acute, incurved; beneath those, the central portion is composed of abundant glandular trichomes, secreting large amounts of sugar crystals (Figs. 5I^′^–5J^′^). From the center to the tip of the mid-lobe, three large, central, verrucose keels, and two or more lateral keels are present (Fig. 5I^′^).

### Subtribe Laeliinae: *Encyclia microbulbon*

***In the intermediate late stage:*** the **anther** is quadrate, with the upper corners rounded. The anther apex is inflected about 135° from the axis of the column, under the **anther cap** two pollinia can be seen; at the anther sides, there are two triangular clinandrium teeth (Fig. 2R^′^). The middle part the rostellum is acute, the adaxial surface has longitudinal elongated cells, in the abaxial side the rostellum is smooth (Fig. 3Y). Below, in the mid part of the **column** a depression with secretions is present (Fig. 2R^′^). In a subsequent stage, the **anther** is more inflected to approximately 160° from the axis of the column, sub-quadrate, with its upper part bi-lobed and the corners rounded. At the apex of the **anther cap** the tip of two pollinia are exposed. The transversal rostellum forms a barrier separating the stigma. Below, a depression with few secretions is observed corresponding to the future stigmatic surface. At the sides on the base of the anther, two triangular clinandrium teeth are present (Fig. 2S^′^). ***Anthesis:*** the apex of the obcordate rectangular **anther** is 170° from the axis of the column (Fig. 2T^′^). In the middle of the upper part of the **anther cap** abundant semi-actinocytic stomata are present (Fig. 4O). Cubic crystals are observed surrounding the stomata (Fig. S1). Below the anther cap four pollinia are visible in a closer view, and two pairs of **caudicles** are exposed (Fig. 6O). The clinandrium has lateral triangular teeth, one on each side of the anther (Fig. 2T^′^). The transverse rostellum is oblong, and the stigmatic surface is seen under it (Fig. 3Z). The **lip** is tri-lobed. The lateral lobes are erect with free tips, the mid-lobe is circular-ovate with crenulated margins, and in the center the surface is verrucose, without keels (Figs. 5K^′^–5M^′^).

## Discussion

### Incumbent anther

Despite the enormous variation in the degree of incumbency or bending of the anther in Orchidaceae, three general pathways of achieving it have been identified, two of them observed in this work. The first pathway described from the distant subfamily Vanilloideae, is the result chiefly of the substantial elongation of the anther connective tissue (Freudenstein, Harris & Rasmussen, 2002); the second pathway is present in the non vandoid epidendroids (where most of this study’s samples are placed), in which incumbency takes place on late ontogeny stages as the result of extension and tipping of the mature anthers (Freudenstein, Harris & Rasmussen, 2002; Valencia-Nieto, Sosa & Márquez-Guzmán, 2016). The third and, the most variable pathway, the so-called Vandoid morphology or syndrome, is observed in the development of *Corallorhiza maculata* and *Govenia alba* of Calypsoinae (a subtribe recently included in Epidendreae) included in this study. In this pathway, bending is reached in early ontogenetic stages, as the result of changes in direction of growth, that lead the anther to begin its development in a starting position with incumbent orientation (called early anther incumbency) and associated to the superposed pollinia (result of a reorientation of the developing thecae), and also to the presence of pollinium stalks or accessory structures of the pollinia, derived from the rostellum (varying zones and amounts of tissue of this structure) which are different in origin to the caudicles (extensions derived from the pollinia) (Freudenstein, Harris & Rasmussen, 2002).

Our micrographs showed that in the members of Bletiinae (including *Chysis* formerly considered in its own subtribe), Ponerinae, Pleurothallidinae and Laeliinae the incumbency is reached out by simple elongation of the anther and tipping in late stages in the ontogeny. The only variation among members of this group was the degree of inflection of the anther (see Table 2). This variation seems to be the result of the morphologies associated with the diverse pollination syndromes in this group, depending on the orientation of the pollinaria needed for successful pollination.

### Implications for classification

Two recent phylogenetic studies, one including representative taxa of all orchid groups (Givnish et al., 2015) and another focused in subfamily Epidendroideae (Freudenstein & Chase, 2015) were the basis for revising the classification of Orchidaceae (Chase et al., 2015). In these studies, *Chysis* was retrieved in a clade as the sister group to subtribe Bletiinae, and thus its members were transferred to this subtribe, whereas previously it was considered in its own subtribe Chysinae (Chase et al., 2015). Based on a number of anther characters observed in this study, such as the shape of the anther cap, the ornamentation of the epidermis of the anther, the form and number of pollinia, linear caudicles, absence of column wings, in addition to characters in the labellum we suggest that further inclusion of species in *Chysis* in molecular phylogenies might help clarify the position of this genus either in subtribe Bletiinae or in its own subtribe Chysinae. If species in *Chysis* are retrieved in Bletiinae the anther characters identified here might be defined as symplesiomorphic characters. However, is *Chysis* is placed in its own subtribe, then the anther characters such as linear caudicles in only one pair, unequal pollinia form, shape of anther cap, absence of column wings and like so the characters of the lip such as the numbers of central an accessory keels (3 o 5) and trichomes in it surface will be the main defining characters of this group.

Freudenstein & Chase (2015) and Freudenstein, Yukawa & Luo (2017) consider *Coelia* a member of subtribe Calypsoinae, although it was previously classified in its own subtribe Coeliinae. Our observations of the column of *Coelia triptera* indicate that early anther incumbency is present, it was already inflected approximately 45°. Freudenstein, Yukawa & Luo (2017) proposed placing *Coelia* in the Calypsoinae, arguing that character transformations that lead to detachable viscidium of the rostellum in this subtribe started from the morphology of *Coelia.* However, our observations show that this genus lacks a cellular pollinium stalk or stipe, as well as superposed pollinia, and the only character of the vandoid syndrome which was observed is the early incumbent anther. Moreover the rest of anther characters are congruent with more advanced Epidendreae: like the presence of granulose caudicles (derived from the pollinium), erect column, and the pollinium number. Furthermore, *Coelia* is the only epiphytic genus in a large group composed by terrestrial geophytes including many leafless and mycoheterotrophic taxa, leaving the question open if indeed it should belong in the subtribe Calypsoinae.

Furthermore, a set of characters that our study found to differ between two genera in subtribe Calypsoinae were observed in *Govenia alba* and *Corallorhiza maculata* and they might have implications in classification. Although both genera are characterized by early anther incumbency, characteristic of the Vandoid syndrome, *Govenia alba*, has a tegula (pollen stalk derived from the column) mainly composed of a strand of granulose cells which connect the viscidium to the pollinium apex. This type of pollinium stalk consists of the dorsal cuticle of the rostellum and it has been considered by Rasmussen (1982), Dressler (1993) and Freudenstein (1994a) a type of stipe opposed to the hamulus present in *Corallorhiza maculata*. Both genera have been classified previously in several subtribes in Epidendroideae, but the position of *Govenia* in Orchidaceae has been the most controversial; Dressler (1981) placed this genus on subtribe Corallorhizinae in tribe Maxillarieae of the extinct subfamily Vandoideae: later, Dressler (1993) transferred *Govenia* (subtribe Goveniinae) to tribe Cymbidieae; Freudenstein (2005e) placed this genus in tribe Calypsoeae but, without indicating relationships with the related *Earina* (Agrostophyllinae) or *Bletia* (Bletiinae) (García-Cruz & Sosa, 2005). Recently, Freudenstein & Chase (2015) and Freudenstein, Yukawa & Luo (2017) placed *Govenia* in subtribe Calypsoinae based on their phylogenetic studies in which this genus resulted the sister group to the remainder genera of the *Corallorhiza* clade.

### Pollination biology

In the very diverse Pleurothallidinae (5100 spp. Karremans, 2016), the majority of pollinators are unknown (Karremans, 2010); however, it has been indicated based on floral morphology that species in this group are pollinated by flies (Pridgeon, Solano & Chase, 2001). In addition, two genera this group were reported as having deceive pollination by pseudo-copulation (Blanco & Barboza, 2005). The plants have minute flowers, with segments with great motility and abundant trichomes and secretions (Pridgeon, Solano & Chase, 2001; Blanco & Barboza, 2005; Jersáková, Johnson & Kindlmann, 2006; Karremans et al., 2015). These characters, were observed in *Stelis ciliaris* and *Specklinia digitale,* which have the smallest flowers studied here, and furthermore they were previously identified by a number of pollination and morphological studies (Damon & Roblero, 2007; Karremans et al., 2013; Ignowski et al., 2015) in which different pollinators besides diptera such like bees were reported.

Karremans et al. (2013) reports the pollination by *Drosophila* in the *Specklinia endotrachys* species group; however, this group has a very different morphology to the species included in this study. The endotrachys group presents species that share a notable reddish-orange color of the perianth parts, especially of the sepals, produce long-lived multi-flowered successive inflorescences, and are larger plants with ten or more flowers open simultaneously, but only one per single inflorescence. Differently, *S. digitale* presents much smaller plants, and inflorescences with 4–10 miniature white-to cream-yellowish flowers, its pollinator is also unknown but is related to the sometimes referred *as Pleurothallis grobyi* Bateman ex Lindl group, where Damon & Roblero (2007) reports flies from the families Cecidomyiidae and Phoridae (plus an unidentified species) as pollinators of *Specklinia marginata* closely related to *S. digitalis* in this species group of *Pleurothallis grobyi*, Damon & Roblero (2007) also mention *S. marginata* was visited by bees of the genus Plebeia. Interestingly, the morphology of the flowers observed in this study is almost identical to his close related *S. marginata,* the phylogenetic relationship between this species in *Pleurothallis grobyi* can probably reflect similarity in its pollinators group.

In Ponerinae, especially in *Isochilus*, flower characteristics are associated with hummingbird pollination (Van Der Pijl, Dodson & Flowers, 1969; Siegel, 2011). In *I. major* we observed that the small tubular flowers are purple (rose and orange colors dominate in other species of this genus), that the lips have gray lines, and pollinia are purple-maroon and when collecting samples we also became aware of the absence of fragrance. In addition, during dissections of *I. major* flowers we discovered a mechanism by which the anther cap comes off (it is joined to the grooved lip by a claw), leaving pollinia exposed or being simply removed by only introducing the dissection needle (which resembles the bird beak) and coming out with pollinaria on its surface, presumably as it happens at the time of pollination by hummingbirds. In the other genera of this subtribe (*Ponera, Nemaconia, Helleriella*) the pollinators are unknown, but flower size, and red and yellow colors (especially in *P. juncifolia*) lines in lip and secretions suggest pollination by small wasps or bees (Soto-Arenas, 2005b).

It has been identified that pollination in Laeliinae, another highly diverse group of Epidendreae (with approximately 1500 spp.), is carried out by diverse pollinators, ranging from Lepidoptera in *Brassavola* and in the diverse *Epidendrum*, to Diptera in some other *Epidendrum*, birds in some *Encyclia* and *Epidendrum*, and predominantly Hymenoptera in most *Laelia*, *Prosthechea*, *Encyclia* and *Cattleya* (Van Der Pijl, Dodson & Flowers, 1969; Borba & Braga, 2003; Van den Berg et al., 2009). In *Laelia* it is well known that flowers emit fragrance to attract the pollinating insects, without nectar or other reward; thus, it is considered as deceptive pollination. Taxa in this group are pollinated by large bees and bumblebees. *Laelia* has been recognized as closely related to *Cattleya* but differs, in number of pollinia (eight in *Laelia* vs. four in *Cattleya*, Halbinger & Soto-Arenas (1997) Borba & Braga (2003)) as reported in this study.

Large *Laelia* flowers are considered as “gullet flowers”, with a lip that constitutes a landing platform for the pollinator, in which the lateral lobes are upturned to enclose the column, forming a tunnel-shaped structure, where the pollinator enters in search of nectar, and when it backs away it touches the stigmatic zone and the pollinarium, depositing or removing the later on its back (Halbinger & Soto-Arenas, 1997). We found in our observations that not only the anther bends, but also that the column bends markedly probably to reach an adequate position for allowing pollinators entering the flowers. In *Laelia speciosa*, Soto-Arenas & Solano-Gómez (2007) reported pollination by bumblebees of the genus *Bombus*. Recently, Peraza-Flores, Carnevali & Van den Berg (2016) segregated some members of *Laelia* into *Schomburgkia*, although *L. speciosa* remains as the type and most basal *Laelia.*

Among taxa in the *Encyclia* alliance Higgins (2003) recorded that species in *Prosthechea* produce fragrances and are pollinated by wasps. In *Prosthechea squalida*, our observations of the lip suggest that abundant secretions are produced to attract pollinators, resulting in a reward-based pollination syndrome. In *Encyclia* and *Oestlundia* pollination is accomplished by Hymenoptera (diverse kinds of bees) (Dressler, 1993; Higgins, 2003). We found the presence of cubic crystals around the actinocityc stomata of the anther cap in *E. microbulbon*, probably offered as attractors to the pollinators.

In Bletiinae, in a number of species in *Bletia* and in most *Hexalectris* and *Basiphyllea,* marked autogamy and cleistogamy has been recorded; some reports of autogamy in *Bletia* highlight the lack of development (partial or total) of the rostellum favouring self-pollination (Van Der Cingel, 2001; Salazar, Chavez-Rendon & Jiménez-Machorro, 2016). In *Chysis* the pollinators are unknown; however, it has been suggested that that due to color and lip characters and fragrant flowers, pollination by large bees or Euglossinii bees might be possible (Soto-Arenas, 2005a; Soto-Arenas & Solano-Gómez, 2007). Our observations showed that in the keels of the lip of *Chysis bractescens* and *C. limminghei* there are abundant trichomes and papillae, probably related with production of fragrances that could be collected by the brushes in hind limbs of bees, especially of the Euglossinii group. Our observation in *Chysis laevis* found that the coalescent anther with the column, the lack of trichomes and papillae on lip keels, the dehiscent pollinia at anthesis and an underdeveloped rostellum (only two vestigial appendages observed) probably allow self-pollination. We suggest that autogamy (by cleistogamy or pseudo-clestogamy) seems to be the common mechanism for fruit production in this taxon.

In Calypsoinae the flowers are small to medium, and diverse pollinators have been reported such like noctuid moths in *Tipularia discolor* (Whigham & McWethy, 1980), bumblebees in *Calypso* (Van Der Cingel, 2001; Tuomi et al., 2015), and in *Cremastra* and *Govenia* pollination is by bees (García-Cruz & Sosa, 2005; García-Cruz et al., 2009), in *Dactylostalix* and *Govenia* hover flies were also reported (Pansarin, 2008); and finally, in *Collarohiza* Epididae flies and mosquitoes were recorded as pollinators (Freudenstein, 2005b). We observed in *Govenia alba* a slight fragrance and guide lines on the lip, the column its pronouncedly bent over itself to accomplish a successful pollination by the incumbence leads to the correct placement of pollinaria on the back of pollinator. In *Corallorhiza maculata,* the lip has a bulging structure on its base forming a channel with abundant pores on its epidermis, and as in *Govenia*, the column is pronouncedly bending inward, but in this genus this character suggests self-facilitation of pollination rather than correct placement of pollinaria on pollinators.

## Conclusions

Our study corroborated that in the subtribes Bletiinae, Ponerinae, Pleurothallidinae, Laeliinae and Chysinae anther incumbency is accomplished by simple elongation and tipping of the anther in the late stages of development. It also confirmed that inflexion of the anther reached by the reorientation of growth in the early ontogenetic stages of the anther, the vandoid morphology, was found in Calypsoinae (excluding *Coelia*).

Our study identified that *Chysis* possesses a number of characters such as the shape of the anther cap, the ornamentation of the epidermis of the anther, the form and number of pollinia, the linear caudicles, and the absence of column wings not shared with the rest of the taxa in subtribe Bletiinae; and therefore, suggesting that its position in this subtribe may need revision. Our observations discovered that *Coelia* only shares the early anther incumbency with Calypsoinae, where it was transferred recently; however, the rest of the morphological characteristics correspond to those of other members of Epidendreae, suggesting also that its position needs to be revisited.

We found characters such like crystals around the actinocytic stomata on the anther cap as well as sugar crystals in Laeliinae; coalescent anther with the column, lack of trichomes and papillae on lip keels, and underdeveloped rostellum in cleistogamously forms of *Chysis laevis*; a mechanism by which the anther cap comes off (it is joined with the grooved lip by a claw) in *Isochilus major*. All of them related to pollination syndromes or reproductive biology.

##  Supplemental Information

10.7717/peerj.4383/supp-1 Figure S1 Cubic crystals on the surrounding of the actinocytic stomata of Encyclia microbulbon

10.7717/peerj.4383/supp-2 Table S1 Catalog collection numbers of specimens included in this study where biological samples were obtained

## Additional Information and Declarations

### Field Study Permissions

The following information was supplied relating to field study approvals (i.e., approving body and any reference numbers): Biological samples of flowers and buds were obtained from plants of the Orchidaceae living collection maintained by the Botanical Garden of the Instituto de Biología, UNAM and the botanical garden “Francisco Javier Clavijero” of the Instituto de Ecología, A.C. (INECOL). We had access to the material under the terms of the scientific permits MX-JB-008-DF and VER-FLO-228-09-09. The Mexican species of Orchidaceae are under special protection (Norma Oficial Mexicana, NOM-059-ECOL-2010, Secretaría de Medio Ambiente y Recursos Naturales, Diario Oficial de la Federación 30 December 2010, Mexico, DF) and the botanical gardens hold individuals of every collected taxon. Data on vouchers is indicated in the Supplementary Material (TS1).

### Data Availability

The following information was supplied regarding data availability: The SEM preparations (cylinders with mounted biological samples gold sputter coated) were just conserved for a year by SEM Laboratory and following their policies, they dismounted it and recovered the gold from preparations for reuse. The biological samples come from plants maintained in the living collections of the Botanical Garden of the Instituto de Biología, UNAM and the botanical garden “Francisco Javier Clavijero” of the Instituto de Ecología, A.C.
