# Supplementary figures and images for: Anther development in tribe Epidendreae: orchids with contrasting pollination syndromes
Source: PeerJ. 2018 Feb 27;6:e4383. doi: 10.7717/peerj.4383 (PMC5833465; doi:10.7717/peerj.4383)

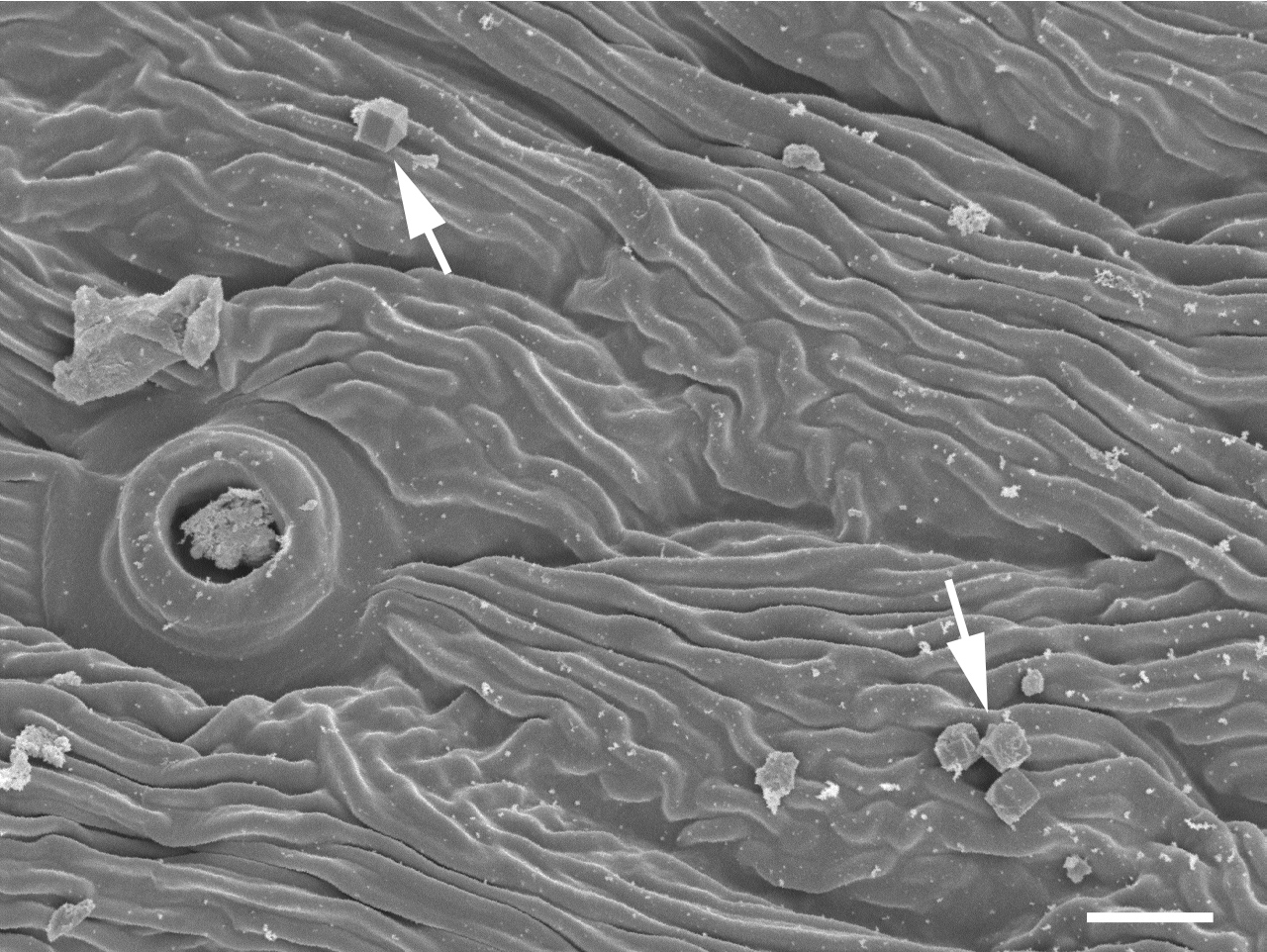

Supplement: Figure S1 [file peerj-06-4383-s001.jpg]
