# Supplementary material for: Anther development in tribe Epidendreae: orchids with contrasting pollination syndromes
Source: PeerJ. 2018 Feb 27;6:e4383. doi: 10.7717/peerj.4383 (PMC5833465; doi:10.7717/peerj.4383)
Supplement: Table S1 [file peerj-06-4383-s002.docx]

**Table S1 Catalog collection numbers of specimens included in this study where biological samples were obtained.**

| **Jardín Botánico IB-UNAM Collection** | **Catalog number** |
| --- | --- |
| *Stelis ciliaris* | **MX-JB-008-DF-8982** |
| *Prosthechea squalida* | **MX-JB-008-DF-8983** |
| *Ponera juncifolia* | **MX-JB-008-DF-8984** |
| *Coelia triptera* | **MX-JB-008-DF-8985** |
| *Chysis laevis* | **MX-JB-008-DF-8986** |
| *Oestlundia ligulata* | **MX-JB-008-DF-8987** |
| *Govenia alba* | **MX-JB-008-DF-8988** |
| *Bletia purpurea* | **MX-JB-008-DF-8989** |
| *Laelia speciosa* | **MX-JB-008-DF-8990** |
| *Chysis bractescens* | **MX-JB-008-DF-8991** |
| *Isochilus mahor* | **MX-JB-008-DF-8992** |
| *Chysis limminghei* | **MX-JB-008-DF-8993** |
| *Specklinia glandulosa* | **MX-JB-008-DF-8994** |
| *Encyclia microbulbob* | **MX-JB-008-DF-8995** |
